# A Pan-*Lyssavirus* Taqman Real-Time RT-PCR Assay for the Detection of Highly Variable *Rabies virus* and Other Lyssaviruses

**DOI:** 10.1371/journal.pntd.0005258

**Published:** 2017-01-12

**Authors:** Ashutosh Wadhwa, Kimberly Wilkins, Jinxin Gao, Rene Edgar Condori Condori, Crystal M. Gigante, Hui Zhao, Xiaoyue Ma, James A. Ellison, Lauren Greenberg, Andres Velasco-Villa, Lillian Orciari, Yu Li

**Affiliations:** Poxvirus and Rabies Branch, Division of High-Consequence Pathogens and Pathology, National Center for Emerging and Zoonotic Infectious Diseases, Centers for Disease Control and Prevention, Atlanta, GA, United States of America; Colorado State University, UNITED STATES

## Abstract

Rabies, resulting from infection by *Rabies virus* (RABV) and related lyssaviruses, is one of the most deadly zoonotic diseases and is responsible for up to 70,000 estimated human deaths worldwide each year. Rapid and accurate laboratory diagnosis of rabies is essential for timely administration of post-exposure prophylaxis in humans and control of the disease in animals. Currently, only the direct fluorescent antibody (DFA) test is recommended for routine rabies diagnosis. Reverse-transcription polymerase chain reaction (RT-PCR) based diagnostic methods have been widely adapted for the diagnosis of other viral pathogens, but there is currently no widely accepted rapid real-time RT-PCR assay for the detection of all lyssaviruses. In this study, we demonstrate the validation of a newly developed multiplex real-time RT-PCR assay named LN34, which uses a combination of degenerate primers and probes along with probe modifications to achieve superior coverage of the *Lyssavirus* genus while maintaining sensitivity and specificity. The primers and probes of the LN34 assay target the highly conserved non-coding leader region and part of the nucleoprotein (N) coding sequence of the *Lyssavirus* genome to maintain assay robustness. The probes were further modified by locked nucleotides to increase their melting temperature to meet the requirements for an optimal real-time RT-PCR assay. The LN34 assay was able to detect all RABV variants and other lyssaviruses in a validation panel that included representative RABV isolates from most regions of the world as well as representatives of 13 additional *Lyssavirus* species. The LN34 assay was successfully used for both ante-mortem and post-mortem diagnosis of over 200 clinical samples as well as field derived surveillance samples. This assay represents a major improvement over previously published rabies specific RT-PCR and real-time RT-PCR assays because of its ability to universally detect RABV and other lyssaviruses, its high throughput capability and its simplicity of use, which can be quickly adapted in a laboratory to enhance the capacity of rabies molecular diagnostics. The LN34 assay provides an alternative approach for rabies diagnostics, especially in rural areas and rabies endemic regions that lack the conditions and broad experience required to run the standard DFA assay.

## Author Summary

Rabies is a preventable disease–but is still responsible for approximately 70,000 human deaths worldwide each year. The majority of human deaths occur in Asia and Africa where there is a lack of diagnostic resources and expertise, making it difficult to develop effective prevention and control strategies. In recent years, several real-time RT-PCR based diagnostic assays have been introduced to many developing countries in an effort to control the H1N1 pandemic flu, Ebola outbreak, and other tropical viral infections. In an effort to further improve rabies diagnostics, we developed a pan-lyssavirus Taqman real-time RT-PCR assay called LN34 for the detection of all known RABV variants and other lyssavirus species. The LN34 assay uses a combination of degenerate nucleotides, multiplex primers and probes, and unique probe modifications to achieve superior sensitivity and specificity compared to previously published RT-PCR based rabies diagnostics. Equally important, the LN34 assay is simple to set up, high throughput, combines multiple standard controls and can be used directly in widely available real-time RT-PCR systems. The LN34 assay was validated using a broad and comprehensive panel of highly diverse RABV variants and other lyssaviruses. A validated universal rabies diagnostic assay will be important in regions where RABV and other lyssaviruses co-circulate and for establishing a widely accepted diagnostic protocol. Over 200 clinical samples (including ante-mortem, post-mortem, and field derived samples) were tested with the LN34 assay, and the assay achieved 100% diagnostic sensitivity and specificity in our laboratory. Over 300 published genome sequences from representatives of RABV and other lyssaviruses were found to contain the conserved LN34 primer and probe targeting sites in an *in silico* analysis. We are expanding the validation of the LN34 assay to multiple domestic and international laboratories and expect the LN34 assay will drastically improve rabies diagnostic capacities globally.

## Introduction

Rabies is an acute progressive viral encephalitis characterized by central nervous system disorder that ultimately leads to death [1]. Rabies is the only disease considered to have nearly 100% mortality, and remains a major public health problem in Asia and Africa with 70,000 human deaths annually, of which approximately 21,000 occur in India alone [2, 3]. Rabies results from an infection by different species of the genus *Lyssavirus*, family *Rhabdoviridae*, with *Rabies virus* (RABV), the type-species of the genus responsible for the majority of deaths [4]. Lyssaviruses are grouped into at least three phylogenetic groups: Phylogroup I (RABV, *Aravan virus* [ARAV], *Khujand virus* [KHUV], *Bokeloh bat lyssavirus* [BBLV], *Duvenhage virus* [DUVV], *European bat lyssavirus* 1 [EBLV-1], *European bat lyssavirus* 2 [EBLV-2], *Australian bat lyssavirus* [ABLV], and *Irkut virus* [IRKV]), Phylogroup II (*Mokola virus* [MOKV], *Shimoni bat virus* [SHIBV], and *Lagos bat virus* [LBV]) and Phylogroup III (*Ikoma lyssavirus* [IKOV], *West Caucasian bat virus* [WCBV] and recently proposed *Lleida bat virus* [LLBV]); and the novel *Gannoruwa bat lyssavirus* species [5–8]. The significant diversity in genome sequences between RABV, especially among lyssaviruses, make it difficult to develop a single, robust, easy to use diagnostic assay.

Currently, a definitive rabies diagnosis can only be confirmed by post-mortem testing. The World Health Organization (WHO) and the World Organization for Animal Health (OIE) have defined the direct fluorescent antibody (DFA) test as the gold standard for rabies diagnosis [9]. The DFA test is a rapid and sensitive method, but its accuracy depends on the quality of brain tissue, availability of high-quality anti-rabies diagnostic conjugates, accessibility to a fluorescence microscope and, most important, an experienced diagnostician [10]. Virus isolation is recommended by OIE in cases of inconclusive DFA results and for detailed molecular characterization of the virus [4]. The laboratory criteria for ante-mortem diagnosis of human rabies include a clinically compatible case that must be confirmed by the detection of lyssavirus antigens, specific antibody, or viral RNA in a clinical specimen at a state or federal public health laboratory [11].

Molecular methods have several advantages in rabies diagnostics compared to other methods, including improved sensitivity, higher throughput, and the potential for variant typing. RT-PCR assays are essential for the detection of *Lyssavirus* RNA in tissues with low viral load such as saliva, nuchal skin biopsy, and eyewashes; moreover, RT-PCR assays can be used to assess the efficacy of experimental therapeutics as well as for risk assessment to prevent nosocomial transmission [12, 13]. In the past, several gel-based conventional RT-PCR assays using simple, nested, or hemi-nested approaches for *Lyssavirus* RNA detection in clinical samples have been published [14, 15]. The amplicons generated by conventional RT-PCR assays require sequencing for confirmation and for phylogenetic analyses. These assays are reported to be highly sensitive when using a hemi-nested approach [16]; however, conventional RT-PCR, especially hemi-nested RT-PCR, is prone to non-specific amplification, leading to false-positive results. An amplicon sequencing step, which is both time consuming and labor intensive, is necessary to rule out potential false positive cases. Real-time RT-PCR based assays have improved sensitivity and specificity compared to other methods, highlighting the potential of a real-time RT-PCR assay to become a leading assay for rabies laboratory diagnosis. Over the last decade many studies have been published assessing real-time RT-PCR assays for the detection of RABV and other lyssaviruses via SYBR Green or TaqMan probe [17–21] methods. TaqMan probe based real-time RT-PCR assays often have lower sensitivity relative to SYBR Green based assays due to the sensitivity of TaqMan probes to the diversity of target sequences in the genomes of different RABV and other lyssaviruses [18, 22–24]. SYBR Green technology based real-time RT-PCR assays may provide greater sensitivity but can carry significant risk of false positive results when performed without a confirmatory sequencing step [20]. Typically, a combination of two or more assays is used to improve assay sensitivity and account for the breadth of lyssavirus diversity [25].

In this study, we investigated the potential of a new real-time RT-PCR assay, which utilizes degenerate, multiplex primers and probes and minor-groove-binding protein (MGB) or locked nucleotide (LNA) modified probes to overcome the limitations of prior real-time RT-PCR rabies diagnostic assays. This new assay is able to detect representatives from all formally accepted RABV variants and all other *Lyssavirus* species with superior sensitivity and specificity, compared to traditional hemi-nested RT-PCR methods.

## Materials and Methods

### Samples

Brain tissue samples from rabid animals were obtained via routine surveillance activity of the Centers for Disease Control and Prevention (CDC; Atlanta, GA, USA). Institutional Animal Care and Use Committee (IACUC) or ethics committee approval was not necessary because moribund animals, dead animals, or animals involved in human rabies exposure were collected by the US State Health Departments, US Department of Agriculture and veterinary laboratories during routine surveillance and diagnostic service. The authors did not perform any animal sampling during this study.

#### Validation panel samples

A total of 88 representatives of RABV and other lyssaviruses were selected for the validation panel. Samples used in the validation panel were derived from known rabies positive animal brain tissue stored in the CDC repository. RNA was extracted from either brain stem or tissue culture supernatant. Viruses were amplified in tissue culture only if limited original material was available. The virus isolates and the details about the host species, geographical location and source of extraction are detailed below.

#### Clinical samples

Human ante-mortem, human post-mortem, and samples from animals that had a history of known human exposure received by the Poxvirus and Rabies Branch (CDC; Atlanta, GA, USA) for testing during November 2014 –November 2015 were defined as clinical samples. A total of 206 clinical samples both from humans (n = 37) and animals (n = 169) were included in this study. All clinical samples were tested by DFA and/or conventional RT-PCR, and the diagnosis and patient treatment recommendations were not based on the results of the LN34 assay.

#### DFA unfit field samples

A total of 58 field samples were tested using the LN34 and β-actin real-time RT-PCR assays. These samples were part of annual surveillance testing (n = 40) supplemented with samples collected by USDA/APHIS/Wildlife services during passive surveillance (n = 18). All samples were deemed unfit for testing by the DFA test due to limited tissue and preservation conditions.

### Development of the LN34 real-time RT-PCR assay

Primer and Probe Design: The LN34 assay amplifies a 165 nucleotide region including the leader sequence, the transcription initiation signal, and part of the coding sequence of the nucleoprotein (N) gene. The forward primers are a multiplex of two oligos that target the beginning of the RABV genome, whereas the reverse primer targets the N gene and contains 6 degenerate nucleotide modifications.

The LN34 probe targets the transcription initiation signal sequence and contains a single degenerate pyrimidine (C or T) at position 65. Probes were modified by either LNA or by MGB. The sensitivities and specificities of the LNA and MGB modified probes were compared in the validation process. Table 1 contains primer and probe sequences and modifications. Degenerate nucleotides are indicated using the IUPAC nucleotide ambiguity code.

**Table 1:** List of primers and probes used in this study.

Name	Length	Sequences ^a^	Position ^b^
Probe LN34	17	(FAM) AA+C+ACCY+C+T+ACA+A+TGGA (BHQ1)	59–75
Probe LN34a	17	(FAM) AAC+ACCYC+T+ACA+A+TGGA (BHQ1)	59–75
Probe LN34lago	17	(FAM) AA +C +ACTA +C +T +ACA +A +TGGA (BHQ1)	59–75
Probe LN34m	17	(6FAM)-ACACCYCTACAATGGAT-(MGBNFQ)	60–76
Primer forward1	24	ACGCTTAACAACCAGATCAAAGAA	1–24
Primer forward2	25	ACGCTTAACAACAAAATCADAGAAG	1–25
Primer reverse	25	CMGGGTAYTTRTAYTCATAYTGRTC	140–164

^a^ The probes are labeled by fluorescent FAM at the 5′end, Black Hole quencher (BHQ1) at the 3′ end except probe LN34m which is labeled by MGB and NFQ quencher. LNA modified bases are indicated by a plus preceding the base in the sequence (e.g. +A, +G, +C, +T).

^**b**^ The primer and probe positions are given relative the *Lyssavirus* full genome (see below).

Two LNA modified probes, LN34 and LN34a, contain a 5′FAM florescent label indicated in parentheses, LNA modified nucleotides indicated by a plus preceding the location of the LNA base in the sequence (e.g. +A, +G, +C, +T), and a 3′ BHQ1 quencher (Table 1, in parentheses). The LN34lago probe is specific for Lagos bat virus lineage B and C subspecies and was mixed with the LN34 probe in a multiplex format; LN34m is an MGB modified probe with a 5′ 6FAM florescent label and 3′ MGB and NFQ quencher.

### LN34 assay controls

An artificial positive control RABV RNA was developed for the assay based on a previous publication [26] and was used to identify potential contamination. The sequence contains 127 bases:

oLPC-rabies3-4: GCA CAG GGT ACT TGT ACT CAT ACT GAT CTG AAT CCA TTG TAG AGG TGT TAG AGC ACG ACA GGT TTC CCG ACT GGA TCT TTC TTT GAT CTG GTT GTT AAG CGT TCG CCC TAT AGT GAG TCG TAT TAC A

A previously described β-actin real-time RT-PCR assay was used in this study, with slight modifications, as an internal or negative control [23].

βactin probe: (HEX)-TCC ACC TTC CAG CAG ATG TGG ATC A-(BHQ1)

β-actin forward primer: CGATGAAGATCAAGATCATTGC

β-actin reverse primer: AAGCATTTGCGGTGGAC

### Optimization

The reaction conditions were optimized for multiple factors, including annealing temperature, length of reverse transcription and PCR reaction steps, as well as the ratio of primers, probes, and master mix components. The optimized reaction conditions are as follows: Ag-Path ID One-Step RT-PCR Kit (Life Technologies) was used with either the LN34 assay primer and probe sets (LN34 and LN34lago) or the β-actin assay primer and probe set. One femtogram (about 10,000 copies) of the artificial RABV RNA template was used as positive control for the LN34 assay. One μl of forward and reverse primer stocks (10 μM) and 1.0 μl of probe (5 μM) were used in the 25 μl reaction set up following the directions of the commercial kit. The cycling conditions are as follows: reverse transcription at 50°C for 30 minutes, followed by RT inactivation / initial denaturation at 95°C for 10 minutes, and amplification for 45 cycles at 95°C for 1 second and 56°C for 20 seconds on a ViiA7 real-time PCR system (Applied Biosystems).

### LN34 limit of detection by droplet digital PCR (ddPCR)

We used the One-step RT-ddPCR advanced kit (Bio-Rad, CA), which delivers improved efficiency and specificity for precise RNA target quantification by Droplet Digital PCR. Details of the reaction set up and cycling conditions are provided in S1 Text.

### Direct fluorescent antibody (DFA) test

All clinical samples were tested for the presence of RABV antigens using the DFA test using fluorescein-isothiocynate (FITC)-conjugated anti-RABV monoclonal antibodies (Fujirebio Diagnostics Inc., Malvern, PA, USA Cat 800-092 and EMD LIGHT DIAGNOSTICS Rabies DFA Reagent Cat 5100) [9].

### RNA extraction and RT-PCR/hemi-nested RT-PCR

Total RNA was extracted from brain, skin, cornea, saliva, or tissue culture supernatant using Trizol reagent (Invitrogen) according to the manufacturer’s instructions. Reverse transcription, amplification of the viral RNA and sequencing of the N gene were performed as described by Hughes et al. 2006 [27]

### *In Silico* and phylogenetic analysis

BioEdit software (http://www.mbio.ncsu.edu/BioEdit/bioedit.html) was used for the alignment of LN34 target sequences. N gene sequencing was performed as described by Hughes et al. 2006 [27]. Eighty-eight whole N gene nucleotide sequences included in the validation panel were subjected to multiple sequence alignments using CLUSTALW (http://www.ebi.ac.uk/clustalw/index.html). All sequences were edited to 1350 bp fragments using BioEdit (S2 Text) [28]. A similarity matrix was calculated using the 88 aligned sequences with MEGA 6 and is summarized in S1 Table [29]. A phylogenetic tree was constructed using the full N gene sequences of the 88 samples in the validation panel and 104 sequences retrieved from GenBank (S1 Fig) using the neighbor-joining method and Kimura-2 parameter substitution model with 1000 bootstrap replicates.

## Results

**Fig 1:**
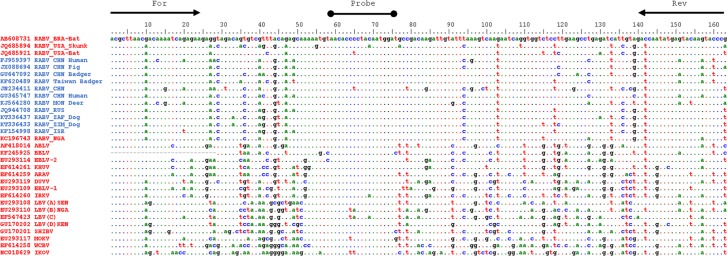
Sequence alignment of the LN34 assay target region from isolates of 14 *Lyssavirus* species.

We sought to design and develop an assay capable of amplifying and detecting a region of the genome conserved across divergent *Lyssavirus* species. The LN34 assay amplifies a 165 nucleotide region including the 58 nucleotide leader sequence, the 12 nucleotide transcription initiation signal, and the first 95 nucleotides of the coding sequence of the N gene (Fig 1). The leader region and transcription initiation signal are strictly conserved in length in all known lyssaviruses [30]. The forward primers target the first 25 nucleotides of the leader sequence with a low level of degeneracy, while the reverse primers use 6 degenerate nucleotides to cover the significant diversity of its target sequence within the N gene. The LN34 probe targets the transcription initiation signal sequence from position 59 to 75 and utilizes a single degenerate pyrimidine (C or T) at position 65. This duplex probe design matches perfectly to all published RABV and other *Lyssavirus* sequences except a few striped skunk RABV variants, MOKV, IKOV, IRKV and LBV subspecies lineages B and C. Fig 1 summarizes the sequence variation in the genomic region corresponding to the probe target sequence from over 200 lyssaviruses.

To compensate the low melting temperature of the 17 nucleotide probes, the probes were modified either by MGB protein (LN34m) or LNA (LN34). The melting temperatures of the modified probes were calculated using either the software Primer Express 3.01 for the MGB probe (Life Technology, California) or the website at http://www.idtdna.com/calc/analyzer for the LNA modified probes (Table 1). Several LNA modified probes containing 5 to 7 LNA nucleotides at different positions of the probe sequence were evaluated using a number of RABV and other *Lyssavirus* samples. Of these, two LNA modified probes (LN34 and LN34a) yielded the best results overall, and the LN34 probe was selected for comparison with an MGB modified probe (LN34m).

The CDC rabies repositories have limited RABV samples from Asia (India and Sri Lanka) and Africa (lineage 1) for the validation process. We used *in silico* analysis to overcome this shortcoming; the sequences of representatives from all major clades or lineages of South-East Asia RABV strains, African RABV lineage 2 and 4 (Fig 1, sequences with blue labels) were analyzed by sequence alignment. As the LN34 assay target sequences are highly conserved or identical among those RABV representatives labeled in blue in Fig 1, LN34 is likely to be able to detect other RABV variants within the same phylogenetic cluster. The global phylogenetic tree in S1 Fig was generated using sequences from 192 RABV and other lyssaviruses. These 192 sequences were chosen based on their comprehensive coverage of *Lyssavirus* species, variants, and geographic locations. The sequences labelled in red correspond to samples that were directly tested in the LN34 assay validation experiments.

A multiple sequence alignment revealed that all the RABV sequences in the global tree have conserved LN34 targeting sequences. The sequences used in this alignment were selected from over 280 published *Lyssavirus* genome sequences in Genbank; only sequences from representatives of RABV and other lyssaviruses that exhibited variation at the probe targeting positions or were representative of unique clades not available in the validation panel are displayed in the sequence alignment. The available BBLV genome sequence lacks the first 29 bp. The available LLBV genomic sequence has limited N gene coverage and is not shown in the sequence alignment. The positions of the forward primer (For), reverse primer (Rev), and probe are listed in Table 1. For the 31 sequences included in Fig 1: samples used in the validation panel are labeled in red with the corresponding Genbank accession number, *Lyssavirus* species, three letter country code and host, if known. Samples that were not available for validation but are representative of unique clades of the global phylogenetic tree (S1 Fig) are labeled in blue.

After initial validation of the LN34 and LN34m probes and parameter optimization using a small set of selected RABV RNA, the LN34 and LN34m probes were evaluated using a panel of RABV samples from the CDC repository. This validation panel (n = 88) was selected to include representative variant samples from around the world (Table 2) including 59 variants of highly divergent RABV isolates from Asia, Africa, Europe, South America and North America.

**Table 2:** *Rabies* virus positive samples used to validate the sensitivity of the LN34 real-time RT-PCR assay.

S. No.	Phylogroup	Isolate Origin	Host	Source	Virus Species	LN34m^	LN34^
1	I	Russia	Dog	TCS	RABV	16.542	ND
2	I	China	Dog	TCS	RABV	28.989	28.895
3	I	Argentina	Dog	TCS	RABV	23.143	23.331
4	I	Thailand	Dog	TCS	RABV	17.425	16.504
5	I	Thailand	Dog	BS	RABV	26.354	24.969
6	I	Thailand	Dog	BS	RABV	21.387	21.522
7	I	Mexico	Dog	TCS	RABV	25.348	25.341
8	I	Mexico	Dog	TCS	RABV	26.509	24.454
9	I	Sri Lanka	Dog	TCS	RABV	22.037	22.427
10	I	Tunisia	Dog	TCS	RABV	22.318	22.089
11	I	India	Dog	TCS	RABV	20.767	19.454
12	I	India	Dog	TCS	RABV	19.801	21.066
13	I	India	Dog	TCS	RABV	25.703	26.135
14	I	India	Dog	TCS	RABV	29.210	28.878
15	I	Gabon	Dog	BS	RABV	27.304	25.406
16	I	Peru	Dog	BS	RABV	18.995	18.427
17	I	Arizona, USA	Bat-Lasiurus spp.	BS	RABV	26.892	27.354
18	I	Arizona, USA	Bat-Lasiurus spp.	BS	RABV	20.186	17.338
19	I	Tennessee, USA	Bat-Lasiurus spp.	BS	RABV	23.068	22.452
20	I	Tennessee, USA	Bat-Lasiurus spp.	BS	RABV	22.096	20.957
21	I	Tennessee, USA	Bat-Lasiurus spp.	BS	RABV	27.265	26.518
22	I	Washington, USA	Bat-Lasionycteris spp.	BS	RABV	25.117	25.164
23	I	Washington, USA	Bat-Myotis spp.	BS	RABV	29.953	28.473
24	I	Washington, USA	Bat-Eptesicus spp.	TCS	RABV	24.808	25.394
25	I	Washington, USA	Bat-Eptesicus spp.	BS	RABV	20.956	19.938
26	I	Arizona, USA	Bat-Eptesicus spp.	BS	RABV	19.187	18.138
27	I	Alabama, USA	Bat-Tadarida spp.	BS	RABV	33.088	30.792
28	I	California, USA	Bat-Antrozous spp.	BS	RABV	21.599	20.024
29	I	Brazil	Bat-Desmodus spp.	BS	RABV	25.617	24.447
30	I	Mexico	Bat-Desmodus spp.	BS	RABV	25.033	23.005
31	I	Texas, USA	Skunk	TCS	RABV	29.524	14.369
32	I	Texas, USA	Skunk	BS	RABV	19.979	18.899
33	I	California, USA	Skunk	TCS	RABV	20.904	21.001
34	I	California, USA	Skunk	BS	RABV	25.387	25.036
35	I	Wisconsin, USA	Skunk	TCS	RABV	19.871	18.374
36	I	South Dakota, USA	Skunk	BS	RABV	32.672	31.012
37	I	Arkansas, USA	Skunk	BS	RABV	30.328	30.465
38	I	Puerto Rico, USA	Mongoose	TCS	RABV	20.715	19.749
39	I	Puerto Rico, USA	Mongoose	BS	RABV	31.710	29.956
40	I	Florida, USA	Feline	BS	RABV	20.403	20.296
41	I	Serbia *	Feline	TCS	RABV	13.443	13.264
42	I	Texas, USA	Coyote	TCS	RABV	17.206	ND
43	I	Texas, USA	Coyote	TCS	RABV	19.801	18.572
44	I	Texas, USA	Coyote	BS	RABV	27.739	27.046
45	I	Alaska, USA	Fox	BS	RABV	30.119	28.494
46	I	Arizona, USA	Fox	TCS	RABV	19.705	19.063
47	I	Arizona, USA	Fox	BS	RABV	29.856	28.819
48	I	Texas, USA	Fox	TCS	RABV	16.542	ND
49	I	Texas, USA	Fox	BS	RABV	30.963	29.303
50	I	Texas, USA	Fox	BS	RABV	20.205	20.470
51	I	Namibia	Lion	BS	RABV	21.102	21.164
52	I	France *	Red Fox	BS	RABV	15.502	15.363
53	I	France *	Red Fox	TCS	RABV	12.039	11.788
54	I	Greece *	Red Fox	BS	RABV	16.859	16.239
55	I	Italy *	Red Fox	BS	RABV	15.963	14.833
56	I	USA	Laboratory strain	BS	RABV	36.497	36.445
57	I	USA *	Laboratory strain	BS	RABV	16.039	13.123
58	I	Europe *	Laboratory strain	TCS	RABV	11.039	11.687
59	I	Georgia, USA	Raccoon	TCS	RABV	17.206	18.570
60	I	Kyrgyzstan	Bat-Myotis spp.	TCS	ARAV	26.036	25.440
61	I	Tajikistan	Bat-Myotis spp.	TCS	KHUV	23.001	23.883
62	I	South Africa	Bat-Miniopterus spp.	TCS	DUVV	36.803	ND
63	I	South Africa *	Bat-Miniopterus spp.	TCS	DUVV	16.722	15.051
64	I	Zimbabwe	Bat-Nycteris spp.	TCS	DUVV	30.741	31.024
65	I	Kenya	Bat-spp.	TCS	DUVV	20.767	19.454
66	I	Australia	Bat-Pteropus spp.	TCS	ABLV	31.025	28.786
67	I	Australia	Bat-Pteropus spp.	BS	ABLV	25.515	23.952
68	I	Europe	Bat-Eptesicus spp.	TCS	EBLV-1	30.484	30.509
69	I	Denmark	Bat-Eptesicus spp.	TCS	EBLV-1	26.640	26.334
70	I	France *	Bat-Eptesicus spp.	TCS	EBLV-1	14.772	14.729
71	I	Europe	Bat-Myotis spp.	TCS	EBLV-2	41.142	38.847
72	I	United Kingdom *	Bat-Myotis spp.	TCS	EBLV-2	18.826	15.930
73	I	Netherlands	Bat-Myotis spp.	TCS	EBLV-2	25.577	24.847
74	I	United Kingdom *	Bat-Myotis spp.	TCS	EBLV-2	16.263	17.978
75	I	Russia	Bat-Murina spp.	TCS	IRKV	23.988	13.290
76	I	Germany *	Bat-Myotis spp.	TCS	BBLV	20.276	19.442
77	II	Zimbabwe	Feline	TCS	MOKV	35.416	27.557
78	II	Zimbabwe	Feline	BS	MOKV	30.130	20.149
79	II	Cameroon	Crocidura spp.	TCS	MOKV	30.726	22.380
80	II	Nigeria	Crocidura spp.	TCS	MOKV	30.770	28.605
81	II	Central African Rep	Lophuromys spp.	TCS	MOKV	41.764	29.034
82	II	Senegal	Bat-Eidolon spp.	TCS	LBV-A	37.012	37.912
83	II	Nigeria	Bat-Eidolon spp.	TCS	LBV-B	ND	26.932
84	II	Central African Rep	Bat-Micropteropus	TCS	LBV-C	ND	22.960
85	II	Kenya	Bat-Rousettus spp.	TCS	LBV-D	23.723	24.441
86	II	Kenya	Bat-Hipposideros	TCS	SHIBV	18.835	17.042
87	III	Russia	Bat-Miniopterus spp.	TCS	WCBV	29.014	26.771
88	III	Tanzania	African civet	TCS	IKOV	43.269	20.916

All 88 samples (except ND [31] = 5) in the validation panel were tested with both the LN34m and LN34 LNA probes in duplicate. Isolate origin refers to the geographical location and/or distribution of the respective sample.

Source of RNA isolation was either TCS (Tissue Culture Supernatant) or BS (Brain stem).

The samples in the validation panel can be divided into 3 phylogroups: Phylogroup I includes *Rabies virus* [RABV], *Aravan virus* [ARAV], *Khujand virus* [KHUV], *Bokeloh bat lyssavirus* [BBLV], *Duvenhage virus* [DUVV], *European bat lyssavirus-*1 [EBLV-1], *European bat lyssavirus-*2 [EBLV-2], *Australian bat lyssavirus* [ABLV], and *Irkut virus* [IRKV]; Phylogroup II includes *Mokola virus* [MOKV], *Shimoni bat virus* [SHIBV], and *Lagos bat virus* [LBV]; and Phylogroup III includes *Ikoma virus* [IKOV] and *West Caucasian bat virus* [WCBV].

* Indicates that the sample was part of the inter-lab testing panel.

**^** Indicates the mean Ct value of duplicate samples detected using either the LN34m or the LN34 LNA probes.

ND: No data; the sample was not tested by this assay.

All the samples in the table are divided into 4 sections by lines; top section contains samples from RABV (Phylogroup I), next section comprises samples from non-rabies lyssaviruses of Phylogroup I, third section comprises samples from Phylogroup II and the bottom section comprises samples from Phylogroup III.

The validation panel also included representatives of 13 *Lyssavirus* species, but did not include LLBV or the recently identified *Gannoruwa bat lyssavirus* species [8]. Both LN34m and LN34 probes were able to detect all variants from the validation panel except the subspecies lineage B and C of LBV (see below) [32]. For the majority of the isolates in the validation panel, the LN34m and LN34 probes demonstrated similar sensitivity, as shown by their similar cycle threshold (Ct) values (Table 2). However, for those isolates with known sequence variations from the probes, the performances of the LN34m and LN34 probes differed considerably. The stripped skunk RABV and IRKV each has a single nucleotide polymorphism (SNP) at position 69 of the probe sequence (Fig 1). Surprisingly, the Ct values produced using the LN34m probe were reduced by at least 10, compared to that of the LN34 probe for one of the Texas skunk isolate (Table 2, S. No 31) and an IRKV isolate (Table 2, S. No 75). This difference in Ct values equates to at least 100 fold difference in sensitivity. Thus, the MGB modified probe is more sensitive to single SNP changes than the LNA probe. Similarly, MOKV and IKOV (Fig 1) each has a single SNP at position 63 that also led to Ct value differences of 8 to14, which also translates to a significant sensitivity loss when using the LN34m probe (Table 2, S. No. 77–80, 88). As the goal of this assay is to attain high sensitivity to detect a broad range of *Lyssavirus* species, the LNA-modified LN34 probe was used for all future validation.

The sequences from lineages B and C subspecies of LBV have two SNPs relative to the probe target sequence, and the validation panel revealed that those subspecies were not detected by either the LN34m or the LN34 probe. Therefore, a specific LNA modified probe (LN34lago, Table 1) was synthesized for the LBV lineage B and C subspecies and combined with the degenerate LN34 probe to make up the LN34 assay. This updated LN34 assay was able to detect all 4 lineages of LBV in our validation panel (Table 2), and the addition of the LBV probe did not affect the sensitivity or the specificity of the LN34 assay.

The multiplexed LN34 assay was able to detect all known positive samples present in the validation panel (Table 2) and exhibited better overall sensitivity and specificity compared to the RT-PCR and real-time RT-PCR assays used previously in RABV diagnostics and validation. Next, the LN34 assay was further evaluated using a thorough comparison with a conventional RT-PCR assay and DFA test using clinical samples. The LN34 assay was able to identify all the rabies positive clinical samples identified by the DFA test (Table 3, n = 13), yielding a sensitivity of 100%. The LN34 assay also correctly ruled out negative clinical samples confirmed by the DFA test and conventional RT-PCR (n = 193), leading to 100% specificity for this assay. Many among those negative samples were initially reported as inconclusive or unable to be diagnosed by DFA due to issues related to reagent quality. The conventional RT-PCR assay, run in parallel with the LN34 assay, produced amplification of products from several of the negative samples that exhibited molecular weights similar to the positive control, but these products were determined to be the results of non-specific amplification by sequencing.

**Table 3:** Positive clinical samples from human (ante mortem and postmortem) and animal (postmortem) specimen.

Source		Lab ID	LN34^	β-actin^
Human	Skin	A15-2217	37.896	20.149
Human	Skin	A15-2460	32.207	19.759
Human	Saliva	A15-2218	32.376	17.307
Human	Saliva	A15-0728	33.310	19.432
Human	Saliva	A15-0729	35.347	21.456
Human	Saliva	A15-2461	32.747	24.4535
Human	Brain	A15-0731	14.003	18.068
Human	Cornea	K1	33.622	ND
Cat	Brain	A15-2206	22.998	19.948
Bat	Brain	A15-0755	16.121	13.888
Dog	Brain	A15-1861	17.017	14.048
Raccoon	Brain	A15-2026	15.098	15.528
Dog	Brain	A15-2190	18.319	16.244

**^** Represents the mean Ct value of the samples run in duplicate by either the LN34 or β-actin control real-time RT-PCR assay.

ND: Indicates that the sample was not tested by this assay.

The LN34 assay performed well on multiple sample types. The negative clinical samples described above included skin biopsies (n = 14) and saliva (n = 15) from patients with neurological symptoms consistent with rabies as well as DFA-negative or inconclusive animal brain samples of varying quality (n = 164). The positive clinical samples included human ante-mortem skin biopsies (n = 2), saliva (n = 4), cornea (n = 1), human post-mortem brain biopsy (n = 1) and animal brain samples (n = 5) (Table 3). Compared to the brain samples, the RABV viral load and/or RNA levels were much lower in the skin biopsy, saliva, and cornea samples, as indicated by their much higher Ct values in both the LN34 and βactin assays.

In addition to the source tissue, the quality of rabies diagnostic samples often varies greatly due to storage conditions. Three ante-mortem samples (Lab ID A15-0728, A15-0729, and A15-0731) were collected from the same patient and had been stored at room temperature for an extended period of time due to a transportation delay. Although these samples did not meet the required standards for rabies testing due to sample condition, the results from the LN34 assay revealed high viral RNA load in the brain biopsy sample (Table 3). Interestingly, all three positive human ante-mortem saliva samples had similar LN34 Ct values, indicating similar viral load or viral RNA quantity, although they were collected in different clinical settings and storage conditions. The LN34 assay was also used to test 58 field samples collected during US annual surveillance testing and passive surveillance by wildlife services that were determined to be unfit for the DFA test due to limited tissue or the unavailability of appropriate conjugates. The LN34 assay revealed that 18 (31.03%) samples were positive (Table 4).

**Table 4:** Positive animal samples from field studies.

Source		Lab ID	LN34^	β-actin^
Arctic Fox	Brain	A15-2672	26.675	25.673
Bat	Brain	A15-2678	17.787	19.149
Arctic Fox	Brain	A16-0742	20.100	20.617
Arctic Fox	Brain	A16-0743	18.553	17.896
Bat	Brain	A15-0283	14.739	ND
Bat	Brain	A15-0284	34.104	ND
Cow	Brain	A15-0287	15.965	ND
Bat	Brain	A15-0396	16.363	ND
Bat	Brain	A15-0399	19.139	ND
Bat	Brain	A15-0401	17.347	ND
Bat	Brain	A15-0407	15.326	ND
Bat	Brain	A15-0409	19.500	ND
Bat	Brain	A15-1903	14.061	ND
Bat	Brain	A15-1905	18.525	ND
Bat	Brain	A15-1907	24.119	ND
Bat	Brain	A15-1908	30.276	ND
Bat	Brain	A15-1910	24.881	ND
Bat	Brain	A15-1912	28.720	ND

**^** Represents the mean Ct value of the samples run in duplicate by either the LN34 or β-actin control real-time RT-PCR assay.

ND: Indicates that the sample was not tested by this assay.

A major concern with diagnostic assay development is the prevention of false diagnoses due to sample degradation or sample preparation error. To minimize these risks, we included two different controls within the multiplex assay: detection of the housekeeping gene β-actin by real-time RT-PCR and a positive control for LN34 assay amplification. The β-actin assay was adapted from a previous publication with minor modifications [23] and was implemented to assess the presence of RNA in the sample. A LN34 negative result requires the β-actin assay to be positive for a negative rabies diagnosis. The β-actin assay utilizes labelling by fluorescent HEX and has same running conditions as the LN34 assay. Thus, a diagnostic sample can be run on the same plate or even in the same well in a multiplex format. An artificial positive RABV control RNA was also adapted from a previous publication [26] with minor modifications: a short LacZ gene sequence was added to allow for differentiating between the artificial RNA and a positive sample, which allows for the easy identification of potential carry-over contamination. The artificial positive control RNA generates a 127 bp amplicon in the LN34 assay that can be differentiated from the 165 bp amplicon produced by RABV and other *Lyssavirus* positive samples on an agarose gel. The artificially generated positive control RNA can be stored for an extended period in a stabilization buffer and can be used as a standard for the comparison of LN34 assay performance between different laboratories and running conditions.

We next examined the sensitivity of the LN34 assay, as one of the key benefits of real-time RT-PCR over other antigen-based diagnostics is increased sensitivity. The efficiency of the LN34 assay was estimated to be greater than 92% by testing serial dilutions of RNA extracted from samples positive for RABV strain CVS11 (93%), DUVV (92%), and LBV (98%). The detection limit of the LN34 assay was measured by droplet digital PCR assay using both artificial positive control RNA and RABV strain ERA; the LN34 assay was capable of detecting a single digit number of RNA copies for both RNA samples (S1 Text).

Lastly, we evaluated the feasibility of using the LN34 assay to detect all known lyssaviruses by *in silico* analysis. The N gene of all samples used in the validation panel were sequenced, and the newly generated sequences were deposited in Genbank (S2 Text). A similarity matrix of N gene sequences among validation panel lyssaviruses was calculated using Mega6 software. The N gene sequences of RABV representatives exhibited approximately 80% or higher nucleotide identity with other RABV sequences. The lowest identity values were found among RABV from southeastern North America, including those isolated from the vampire bat of Mexico and the raccoon and South Central skunk of the US. This finding is in agreement with previous RABV variant classifications that suggest American indigenous RABV are one of the most divergent strains [30]. The N gene sequences of the examined representatives from non-RABV lyssaviruses had lower nucleotide identity values ranging from 68% to 79%. Also, according to this analysis, the most divergent *Lyssavirus* was IKOV, which is also in agreement with previous studies [6].

The combined sensitivity and robustness of the LN34 assay to detect lyssaviruses from all available species highlights its utility as a diagnostic tool applicable for laboratory rabies diagnosis.

## Discussion

Real-time RT-PCR based diagnostics are not currently recommended by international agencies for routine diagnosis of RABV, but considerable developments in both PCR platforms and PCR chemistry have made this technology increasingly attractive for decision making in rabies case management. The LN34 assay represents a major improvement over previously published real-time RT-PCR assays for the detection of RABV and other lyssaviruses, as it can detect a broad range of phylogenetic diversity with superior sensitivity and specificity. The LN34 assay has shown equal (or possibly better) specificity and sensitivity to the DFA test, and the one step reaction format reduces the chances of false positive results that are a risk inherent to two step and SYBR Green based RT-PCR assays. Also, through implementing strict quality control procedures (i.e., the β-actin internal control and artificial positive control), one can identify and reduce both false negative results in degraded samples and carry-over contamination from positive controls [26].

Initial assay development for the LN34 assay was based on more than 280 published full genome sequences from lyssaviruses of all known species (except LLBV), covering all major geographic based clades: American indigenous, Indian, South-East Asian, African lineage 2, African lineage 3, Arctic-related and cosmopolitan strains. The LN34 forward primer consists of degenerate nucleotides near the 3′ end as the first 9 nucleotides of the leader sequence are identical among all examined lyssaviruses; the reverse primer also targets a relatively conserved region of the N gene that has been used for the design of conventional rabies RT-PCR primers previously [19]. The reverse primer used in the LN34 assay is longer and uses more degenerate nucleotides compared to those used in previous studies in an attempt to maximize the universal amplification from divergent lyssaviruses while maintaining a high level of specificity [19]. The sequence of the LN34 probe targets one of the most conserved regions across individuals from all species of lyssaviruses. A well-known rabies RT-PCR primer (JW12) was developed from the same region as the LN34 probe [15], but the LN34 probe is shorter than JW12 and has identity with most known RABV sequences when a single degenerate site is accounted for (excluding some stripped skunk RABV variants). Additionally, genomic sequences from representatives of at least 8 lyssaviruses, which are more diverse than RABV isolates, exhibited 100% nucleotide identity with the LN34 probe (Fig 1) and could be amplified efficiently using the LN34 primers (Table 2). The low level of sequence degeneracy designed in the LN34 primers and probes allows the LN34 assay to maintain an optimal degree of sensitivity and specificity for rabies diagnostics.

Previous studies have shown that LNA and MGB probes have similar sensitivities [33]. Our evaluation of the MGB and LNA modified LN34 probes revealed similar sensitivities for most of the RABV and other lyssaviruses tested, which agrees with sequence alignments showing that probe target sequence is identical in most of the validation panel samples. However, we observed differences in the sensitivities of the two probes for a few variants with genomic sequence variations that did not correspond to the probe sequence (Fig 1). The LN34 MGB probe was very sensitive to single nucleotide changes in the probe’s target sequence, whereas the LN34 LNA probe exhibited a higher tolerance to single nucleotide mismatches. To allow for detection of a broader range of lyssaviruses, the LNA-modified probe was implemented in the LN34 assay. It remains unclear if the differences in probe tolerance to mismatches is specific to the LN34 probe or it if may affect other probe designs.

The LN34 assay was validated using a large and highly diverse panel containing representative members of major RABV variants and 13 other lyssaviruses. The LN34 Ct values corresponding to all RABV variants tested were highly positive even when samples in the validation panel were diluted 10 fold from the original samples. Since LBV lineage B and C subspecies could not be detected by the initial probe design, a multiplex format containing a specific probe for this subspecies was developed to cover this detection flaw. The additional probe did not affect assay performance, suggesting that the LN34 assay can accommodate changes as novel lyssaviruses emerge. In fact, the newest *Lyssavirus* LLBV, phylogenetically grouped with IKOV (Phylogroup III), was not validated in the LN34 assay [6] because the available sequences do not cover the LN34 probe region and, thus, could not be evaluated in an *in silico* analysis. However, as LLBV sequences and samples become available, a LLBV specific probe can be added to the LN34 assay if the region contains polymorphisms.

The average Ct values of samples in the validation panel were 24.490 and 22.886 using the LN34 and LN34m probes, respectively, after a 10 fold dilution of most RABV samples, testing both brain and cell culture samples. The samples from the annual inter-lab testing 2015 panel, had the lowest Ct values. The range of Ct values observed was most likely due to differences in sample quality, as some of the samples in the validation panel had been frequently used and have degraded over time. Some viral strains, i.e. DUVV and EBLV-1, were prepared in different batches or at different times and varied considerably in their LN34 Ct value (Table 2). For those samples where Ct values differed by more than 3, the samples were re-run on the same plate, and only RABV isolates from Gabon Africa (Table 2 S. No. 15) and a bat associated RABV from the US (Table 2, S. No. 18, 27) still exhibited Ct value differences greater than 2, which may be due to variations in the sequences targeted by the primers. We also noticed slight variations in Ct values produced by the LN34m and LN34 probes, which were likely caused by the assays being completed independently in separate batches.

A key benefit of RT-PCR based assays is the ability to detect limiting quantities of substrate, which has large implications when clinical sample quality and quantity are sub-optimal. Our analysis revealed that the sensitivity of the LN34 assay was consistently superior or equivalent to that of the conventional hemi-nested RT-PCR assay for multiple validation panel samples, although not all samples were tested. Based on the absolute quantification using ddPCR and taking into account assay efficiency, the LN34 assay was able to detect a single digit copy of the RNA template from rabies clinical samples. As the majority of available RABV and other *Lyssavirus* genomes have sequences identical to those of the LN34 probes and primers, the amplification efficiency and the limit of detection of the LN34 assay should remain relatively consistent for most lyssaviruses.

The high sensitivity of the LN34 assay is important for the diagnosis of samples that have been stored or transported under sub-optimal conditions and cannot to be diagnosed by other methods. The standard protocol for DFA testing requires that samples be maintained under cold chain conditions, which is not always feasible. Diagnosis based on ante-mortem samples is particularly challenging, as viral shedding is not only low but is also intermittent; frequent monitoring is necessary, which can be rapidly facilitated using the LN34 assay. In this study, the LN34 assay proved capable of diagnosing several samples that were considered unfit for DFA testing or that were inappropriate for analysis by the DFA test, including surveillance samples as well as multiple ante-mortem samples, despite relatively low level of rabies RNA. All LN34 positive ante-mortem samples were confirmed by direct sequencing of the amplicons produced in the LN34 assay, which could be used to generate genotyping results for the diagnostics.

Each year, rabies surveillance in the United States involves testing over 100,000 suspected rabid animal samples, of which over 6,000 rabid animals were diagnosed in 2014 [34]. The inherent high throughput nature of the LN34 assay has the potential to improve the speed of testing and reduce the workload for diagnostic laboratories. Furthermore, for those thousands of samples unfit for DFA testing due to limited sample quantity or sample degradation, the LN34 assay can be used as the diagnostic test of choice, since it requires very little material. We tested a total of 58 samples considered unfit for DFA testing. Of these, 18 samples (31%) were positive by the LN34 assay (Table 4). By applying the real-time RT-PCR LN34 assay, the number of samples unsuitable for testing can be further reduced, which will improve the surveillance and control of rabid animals in the US.

Current rabies sample collection and storage methods can be tedious, using real-time RT-PCR based diagnostic methods such as the LN34 assay can reduce the burden of sample collection, transportation, and storage for rabies diagnostics and surveillance. This is of particular importance in rural areas or developing countries which often have limited resources and lack experienced laboratorians. Further, nucleic acid based detection techniques are comparatively easier to perform and the technology is deployable to be used in the field. Since this technique has the potential to be used as a high throughput assay, it can thus can be used as a tool for real-time rabies surveillance to check the spread of the virus in wildlife species as well to monitor the usefulness of control programs like oral rabies vaccination. The LN34 assay allows for testing samples that have been stored in stabilization buffers, which means that clinical samples can be stored for extended periods of time under less stringent conditions and significantly reduces the need for cold chain and its associated costs. Furthermore, the collection method can be modified or simplified to reduce the potential for contamination as well as mitigate the risk to the person obtaining the sample; for example, the implementation of a real-time RT-PCR assay would allow for use of the OIE approved rapid sample collection of brain samples which avoids the process of opening the skull and collecting the sample from the occipital foramen (which is particularly challenging in large animals) [35]. Additionally, the artificial RNA control can be produced in large quantities using commercial kits and stored for an extended time using an RNA storage buffer, and the RNA control can be distributed to different laboratories to monitor the performance of the LN34 assay and improve quality control for rabies diagnostics.

WHO and OIE set a goal for canine rabies eradication by 2030; a key component for the success of this goal is to improve rabies diagnostics and surveillance, especially in developing countries. LN34 assay is able to overcome many hurdles for rabies diagnostics in resource limited areas: it reduces the logistical burdens associated with the rabies sampling process, storage, and transportation and the quality of test standards and reagents can be quickly established across laboratories around the world, as the LN34 assay can leverage the current real-time RT-PCR platforms and skills in most laboratories with limited additional training required. Our validation results and the preliminary results in a pilot program implementing the LN34 assay in multiple laboratories show that the LN34 assay increases the accuracy of rabies diagnostics. We expect that the LN34 assay will improve rabies diagnostic capacities in many laboratories around the world.

## Supporting Information

S1 Text Limit of detection using droplet digital RT-PCR. (DOCX)

S2 Text Complete N gene sequences of the samples in the validation panel. (TXT)

S1 Table Similarity matrix of the samples in the validation panel. (XLS)

S1 Fig A global phylogenetic tree of lyssaviruses. 192 N gene sequences (Genbank accession number is used as the identifier for each sample) were used to generate a global phylogenetic tree which represents the geographic diversity of known major RABV variants/lineages and other lyssaviruses (neighbor-joining (Kimura-2 parameter)). The accession numbers labelled in red indicate the samples used in the validation panel (Table 2, Fig 1); the accession numbers labelled in blue represent the samples used in the *in silico* analysis of LN34 assay primers/probe targeting sequences (Fig 1). The phylogenetic clades are labelled at multiple levels: isolate geographic location, major variant/lineage, and phylogroup.
(JPG)
