# Supplementary material for: A Pan-Lyssavirus Taqman Real-Time RT-PCR Assay for the Detection of Highly Variable Rabies virus and Other Lyssaviruses
Source: PLoS Negl Trop Dis. 2017 Jan 12;11(1):e0005258. doi: 10.1371/journal.pntd.0005258 (PMC5230753; doi:10.1371/journal.pntd.0005258)
Supplement: S1 Text — (DOCX) [file pntd.0005258.s001.docx]

Reaction setup:

1. Prepare the reaction mix for the number of reactions needed according to the table below:

Component Volume per final concentration

reaction,ul

Supermix 5ul 1X

Reverse transcriptase 2ul 20U/ul

300mMDTT 1ul 15mM

Target Forward primer 34F 10uM 2ul 1000nM

Target Reverse primer 34R 10uM 2ul 1000nM

Target probe 34P 2.5uM 1ul 125nM

RNase-/DNase-free water 2ul

15ul mix them and then add:

DNA (different dilution) 5ul variable

Total: 20ul

1. Mix thoroughly and load 20ul of each reaction mix into a sample well of a DG8 cartridge for QX100 droplet generator followed by 70ul of droplet generator oil for probes into the oil wells. And then put the DG8 cartridge into droplet generator for 2min.

Thermal Cycling Conditions:

1. After droplet generation with the QX100 droplet generator, carefully transfer droplets into a clean 96-well plate. Heat seal the plate with foil by the PX1 PCR plate Sealer before thermal cycling on an ABI GeneAmp 9700. The cycle conditions see below. The ramp rete setting of the thermal cycle were lowered to 3^0^C/s to allow thorough heating and cooling of the droplets.
2. After PCR, the 96-well plate was placed into a plate holder and loaded into the QX100 droplet digital reader for detection and analysis.

Cycling Conditions:

Cycling step temperature, ^0^C time number of cycles

Reverse transcription 45^0^C 60min 1

Enzyme activation 95^0^C 10min 1

Denaturation 95^0^C 30sec 40

Annealing/extension 55^0^C 1min 40

Enzyme deactivation 98^0^C 10min 1

Hold 4^0^C

Results:

| RABV strain ERA RNA dilutions | LN34 qPCR CT Value | Estimated RNA copies/ul by ddPCR |
| --- | --- | --- |
| 1:25 | 31.15 | 89.8 |
| 1:125 | 34.3 | 15.8 |
| 1:625 | 36.48 | 3.66 |
| 1:3125 | 36.99 | 1.38 |
| 1:15625 |  | 0.39 |
| 1:78125 |  | 0.18 |
| LN34 positive control RNA dilutions | LN34 qPCR CT Value | Estimated RNA copies/ul by ddPCR |
| 1: 390625 | 28.82 | 627 |
| 1:1953125 | 31.51 | 121 |
| 1:9765625 | 33.84 | 31.2 |
| 1:48828125 | 36.43 | 3.11 |
| 1:244140625 | 40.59 | 0.94 |
| 1:1220703125 |  | 0.28 |
|  |  |  |
