# Supplementary figures and images for: A Pan-Lyssavirus Taqman Real-Time RT-PCR Assay for the Detection of Highly Variable Rabies virus and Other Lyssaviruses
Source: PLoS Negl Trop Dis. 2017 Jan 12;11(1):e0005258. doi: 10.1371/journal.pntd.0005258 (PMC5230753; doi:10.1371/journal.pntd.0005258)

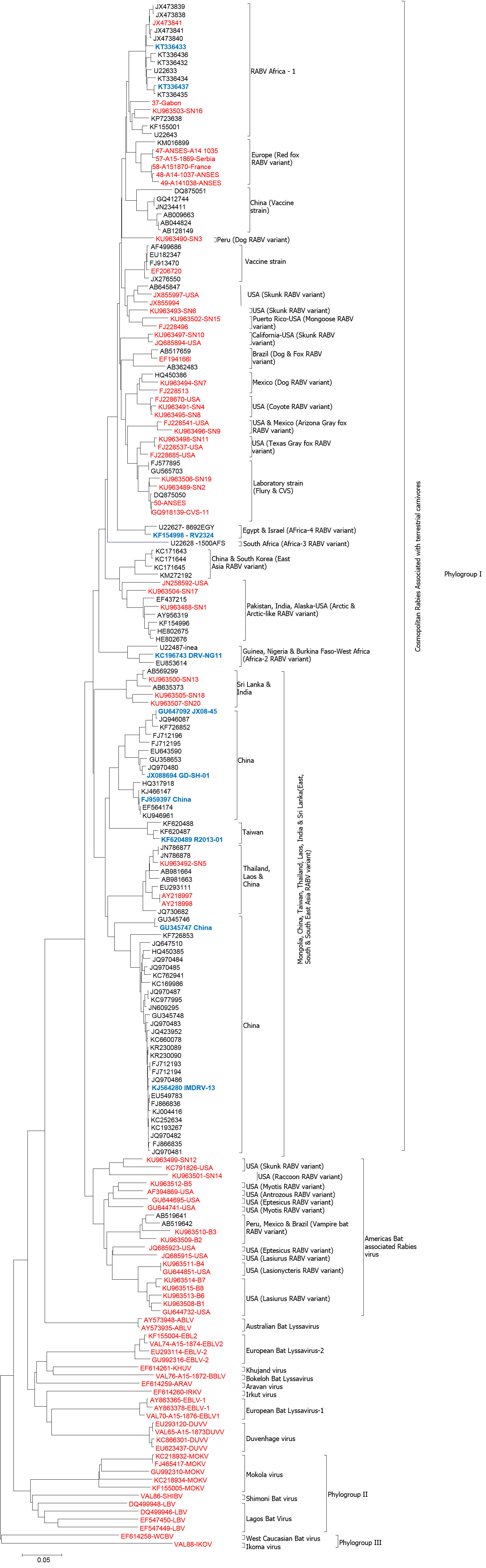

Supplement: S1 Fig — 192 N gene sequences (Genbank accession number is used as the identifier for each sample) were used to generate a global phylogenetic tree which represents the geographic diversity of known major RABV variants/lineages and other lyssaviruses (neighbor-joining (Kimura-2 parameter)). The accession numbers labelled in red indicate the samples used in the validation panel (Table 2, Fig 1); the accession numbers labelled in blue represent the samples used in the in silico analysis of LN34 assay primers/probe targeting sequences (Fig 1). The phylogenetic clades are labelled at multiple levels: isolate geographic location, major variant/lineage, and phylogroup. (JPG) [file pntd.0005258.s004.jpg]
